# Non-Coding RNA-Dependent Regulation of Mitochondrial Dynamics in Cancer Pathophysiology

**DOI:** 10.3390/ncrna9010016

**Published:** 2023-02-20

**Authors:** Maria Eugenia Gallo Cantafio, Roberta Torcasio, Giuseppe Viglietto, Nicola Amodio

**Affiliations:** 1 Department of Experimental and Clinical Medicine, University Magna Graecia of Catanzaro, 88100 Catanzaro, Italy; 2 Laboratory of Cellular and Molecular Cardiovascular Pathophysiology, Department of Biology, Ecology and Earth Sciences (DiBEST), University of Calabria, Arcavacata di Rende, 87036 Cosenza, Italy

**Keywords:** miRNA, long non-coding RNA, mitochondrial dynamics, Drp1, mitochondrial fission, mitochondrial fusion

## Abstract

Mitochondria are essential organelles which dynamically change their shape and number to adapt to various environmental signals in diverse physio-pathological contexts. Mitochondrial dynamics refers to the delicate balance between mitochondrial fission (or fragmentation) and fusion, that plays a pivotal role in maintaining mitochondrial homeostasis and quality control, impinging on other mitochondrial processes such as metabolism, apoptosis, mitophagy, and autophagy. In this review, we will discuss how dysregulated mitochondrial dynamics can affect different cancer hallmarks, significantly impacting tumor growth, survival, invasion, and chemoresistance. Special emphasis will be given to emerging non-coding RNA molecules targeting the main fusion/fission effectors, acting as novel relevant upstream regulators of the mitochondrial dynamics rheostat in a wide range of tumors.

## 1. Introduction

Mitochondria are semi-autonomous dynamic organelles existing in eukaryotic cells, surrounded by a double plasma membrane, with their own genome and protein synthesis apparatus. They deal with several biological functions, including intracellular energy production, cellular metabolism, growth signaling, cell proliferation and differentiation, and cell death [1,2]. The mitochondrial structure consists of four basic elements: (*i*) an outer mitochondrial membrane (OMM), containing three integral protein families: TOMM complex, SAM complex, and porins; (*ii*) an inner membrane space (IMS) that lies between the two membranes; (*iii*) an inner mitochondrial membrane (IMM) with numerous folds, namely cristae, that are the sites for electron transport chain (ETC) assembly and oxidative phosphorylation (OXPHOS); and (*iv*) the mitochondrial matrix (MMX), where the tricarboxylic acid (TCA) cycle takes place [3,4]. Mitochondria have their own DNA, called mitochondrial DNA (mtDNA), that exists in the matrix as thousands of copies of circular DNA [5].

Among the various functions of mitochondria, the most relevant is represented by the energy supply under ATP form. To accomplish it, mitochondria incorporate various metabolic pathways, including TCA cycle, fatty acid oxidation (FAO), amino acid oxidation, OXPHOS, and many others [6,7].

Mitochondria participate in relevant physiological processes, such as Ca^2+^ homeostasis, redox homeostasis, and synthesis of heme and iron-sulfur clusters; moreover, they are tightly associated with apoptosis induction through cytochrome *c* release via the intrinsic pathway, which relies on the mitochondrial outer membrane permeabilization (MOMP) [8].

To facilitate the maintenance of mitochondrial integrity and homeostasis, mitochondria do not work as individual organelles, but as a network that defines the so-called “mitochondrial dynamics”, overall consisting in changes of their number and morphology through the coordinated processes of fusion and fission [9]. 

In this review, we will analyze the involvement of the mitochondrial dynamics process in tumor onset and progression, with a specific focus on its regulation by non-coding RNAs.

## 2. Mitochondrial Dynamics Biology

Mitochondrial fusion and fission are vital physiological mechanisms for the cell, as they can help mitochondria to regulate cellular ATP levels and minimize the build-up of mtDNA damage throughout aging.

The mitochondrial dynamics process is mediated by guanosine triphosphatases (GTPases) of the dynamin family [10,11]. This process consists in the fusion between two mitochondria at the level of OMM, which is mediated by members of the membrane-anchored dynamin family known as Mfn1 (Mitofusin 1) and Mfn2 (Mitofusin 2). Mitofusins co-localize at the OMM, and their GTPase domain is required to pull together the two opposing OMMs, resulting in bilayer fusion between adjacent mitochondria; the fusion between the inner mitochondrial membranes is instead mediated by a single member of the dynamin family called Opa1 [10,12].

Opa1 is considered an indicator of the bioenergetic state, and its activation state depends on mitochondrial ATP levels. Opa1 undergoes proteolytic processing to produce two distinct isoforms (L-Opa1, S-Opa1) that are critical to initiate fusion and maintain cristae junctions [13]; they interact with Mfn1 and Mfn2, and then form a bridge between OMM and IMM, necessary to mix lipids while melting [12]. Lower ATP concentration leads to the cleavage of Opa1 into larger isoforms that favor fusion, while higher levels of ATP break down Opa1 into smaller isoforms that support fission [14].

Conversely, fission (or fragmentation) consists of a division of mitochondria into particles of irregular size, which acts together with mitophagy or apoptosis, facilitating the removal of damaged mitochondria as well as the mtDNA redistribution and mitochondrial mobility [8].

Mechanistically, mitochondrial fragmentation begins with the binding of the enzyme GTPasic Dynamin-Related Protein-1 (Drp1), encoded by the DNM1L gene (human chromosome 12p11), that contains four domains: an N-terminal GTPase domain, a middle domain, a variable domain (or B-insert), and the GED in C-terminal. It is regulated by a range of post-translational modifications, including phosphorylation, ubiquitination, sumoylation, and nitrosylation; other proteins, such as MFF (mitochondrial fission factor, i.e., C2orf33, human chromosome 2q36.3), MID49 (human mitochondrial dynamics proteins 49, i.e., MIEF2, human chromosome 17p11), MID51 (i.e., MIEF1, human chromosome 22q13), and Fis1 (mitochondrial fission 1 protein, i.e., TTC11, human chromosome 7q22), together with the two MIEF1/2 adapters, are crucial to recruit Drp1 that, upon phosphorylation at Ser616, translocates to the OMM, where it binds these adaptor proteins and undergoes conformational changes [9,10,12]. In this way, the Drp1 GTPase domain protrudes from the OMM and connects to other proteins on adjacent mitochondria. Once the Drp1 helix has spiralized around the mitochondria, GTP hydrolysis causes constriction of the helix and scission of the OMM and IMM. MFF is a crucial factor in fission, as its knock-down leads to an elongated mitochondrial network. In addition, MFF physically interacts with Drp1 both in vitro and in vivo, affecting Drp1 function in mitochondrial fission and apoptosis. In contrast, these effects have not been observed for other proteins, including Fis1, thus confirming that MFF is the main recruiter of Drp1 during fission [15,16]. Fis1 participates in the assembly, spatial distribution, and function of fission complexes on the external mitochondrial membrane [16]. MID49 and MID51 are additional Drp1 recruiting proteins at sites where mitochondria come into contact with endoplasmic reticulum [10].

A fine balance between mitochondrial fusion and fission is needed for cell survival and optimal functioning, because mitochondrial dynamics helps maintaining the mitochondrial pool in a cell and optimal OXPHOS activity by allowing for efficient transport and distribution of mitochondrial content, and is ultimately linked to the cell cycle, apoptosis, cell migration, mitophagy, and reactive oxygen species (ROS) production [9]. Of note, the mitochondrial dynamics process has emerged as a cellular defense mechanism. Mitochondria are the main producers of reactive oxygen species (ROS), a byproduct of oxidative phosphorylation that, if exceeded, damages cellular components such as proteins, lipids, and DNA. Indeed, a balanced ROS production is essential to maintain the correct functioning of the cell. An excessive amount of ROS leads to the loss of respiratory chain functional complexes, resulting in a lower bioenergetic capacity that triggers cell death. In this context, mitochondrial dynamics can help compensate damage with the fusion and eliminate it by fission, acting as a defense mechanism, because the reunification of the mitochondrial network can compensate the damage—but only if it is minor [17]. In the case of severely damaged mitochondria, the defense mechanism that kicks in the cell is the autophagy, which consists in damaged organelles elimination through autophagolysosomal incorporation. Conversely, the elimination of damaged mitochondria is called mitophagy, and it has been observed that mitochondrial dynamics may regulate such a process, and specifically the inhibition of fission can trigger mitophagy [10,14,18].

**Figure 1:**
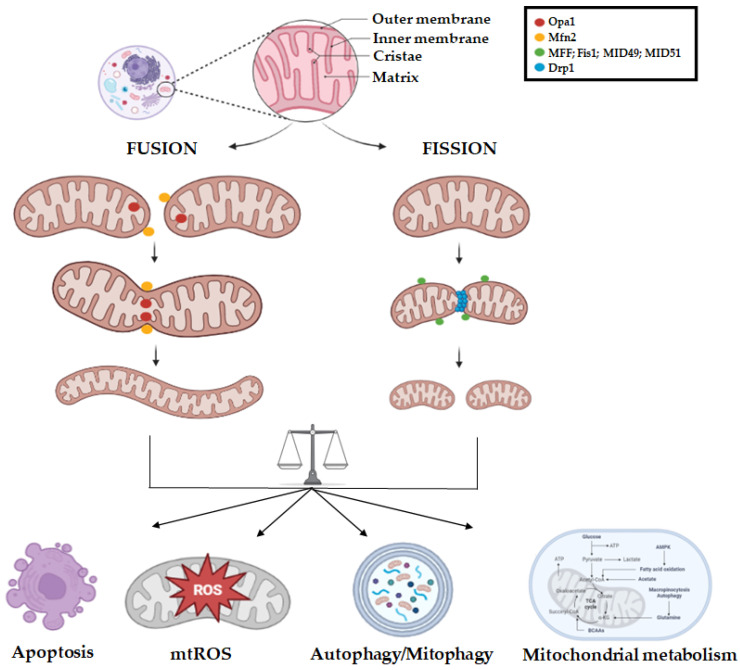
Mitochondrial dynamics machinery. Representative illustration of the mitochondrial fission and fusion events regulated by the main GTPase effector proteins. The fusion of outer mitochondrial membrane is regulated by Mfn1 and Mfn2, whereas the mitochondrial inner membrane fusion, mitochondrial cristae integrity, and remodeling are regulated by Opa1. Drp1 regulates mitochondrial fragmentation by its interaction with the fission receptors MFF, Fis1, MID49, and MID51. A fine balance of fission/fusion processes controls apoptosis, mitochondrial ROS (mtROS) production, autophagy, mitophagy, and mitochondrial metabolic pathways. The picture was created using BioRender software.

When every defense mechanism fails, apoptosis occurs. Mitochondrial apoptosis consists of an external membrane permeabilization that allows the release of cytochrome c, after pro-apoptotic stimuli, from the intermembrane space to the cytosol, arising the intrinsic apoptotic pathway. In this context, it has been reported that the main regulators of mitochondrial dynamics, Mfn1/2 and Drp1, interact with pro-apoptotic proteins, Bak and Bax, thus underscoring a key role of dynamics in apoptosis [19,20]. A graphic overview of the mitochondrial dynamics network is provided in Figure 1.

High levels of cellular stress, leading to apoptotic cell death, can increase mitochondrial fission; in this regard, inhibition of mitochondrial fission can delay the release of cytochrome c, indicating that fragmentation participates in permeabilization of the mitochondrial membrane mediated by Bax [10,14,19].

Another role of mitochondrial dynamics has been observed in cell migration: several studies carried out on breast, thyroid, and glioblastoma tumor cells have shown that mitochondrial fission is necessary to sustain the migration and invasion potential [21,22]; accordingly, downregulation of Drp1, or overexpression of Mfn1 or Mfn2, can antagonize such effects [12]. However, the onco-modulatory role of mitochondrial dynamics is highly tumor-specific, as further discussed in the next sections.

## 3. Mitochondrial Dynamics Aberrations in Cancer

Accumulating evidence demonstrates that mitochondria-driven metabolic reprogramming is involved in cancer development and progression [23,24]. Mutations in both coding and non-coding regions of mitochondrial DNA (mtDNA) have been detected in various cancer types [24,25]; moreover, alterations in the mitochondrial quality control system, which includes pathways coordinating biogenesis, mitophagy, fusion, and fission, unbalance the mitochondrial homeostasis and function [26,27,28], contributing to tumorigenesis.

Since mitochondrial fusion and fission dynamically affect mitochondrial morphology and make energetic adjustments according to the metabolic needs, it is not surprising that dysregulated mitochondrial dynamics have been found associated to cancer pathophysiology.

Besides providing proliferative advantage, metastatic ability, and cell death evasion, oncogenic signaling pathways aberrantly activated in cancer due to mutations and/or epigenetic alterations can affect the mitochondrial dynamics rheostat [29]. The phosphoinositide 3-kinase (PI3K)/protein kinase B (AKT) oncogenic signaling pathway, involved in cell survival/proliferation and differentiation in a wide variety of tumors, can phosphorylate and activate Drp1, while the calcium/calmodulin-dependent kinase II (CAMKII) phosphorylates Drp1 on its inhibitory S637 residue, promoting mitochondrial fission [30]. Mutations in the MAPK signal transduction cascade is a common event in several cancer types [31]. Drp1 is phosphorylated and activated by ERK, leading to enhanced mitochondrial fission, which fosters tumor growth [32]. This fission phenotype seems to fulfill to the need of highly proliferative tumor cells for equal mitochondrial distribution during cell division.

Vemurafenib is a BRAF inhibitor that has significantly changed the therapeutic landscape in melanoma, prolonging the survival of patients with unresectable metastatic or BRAFV600E-mutated melanomas. BRAFV600E-mutated melanoma cells under vemurafenib treatment shows hyperfused mitochondria [33], as a result of both the inhibition of Drp1 S616 phosphorylation and the increased expression of Mfn1/2. Moreover, given that mitochondrial fusion is dependent on high ΔΨ, the OXPHOS impairment and energy exhaustion triggered by respiratory chain inhibitors or uncouplers can further enhance vemurafenib cytotoxicity.

There is nowadays robust evidence that tumor cells modify the mitochondrial dynamics rheostat in order to gain proliferative and survival advantages. Increased mitochondrial fission has been reported in several types of human cancer cells (melanoma, ovarian, breast, lung, thyroid, glioblastoma, and others), with a clear correlation with the onset of chemoresistance. Mitochondria are found in a fragmented pattern within different types of tumor cells, caused by Drp1 upregulation [34]. Such enhanced mitochondrial fission causes metabolic reprogramming, cell cycle progression, and increased migration, invasiveness, and metastatic potential. 

The selective antagonism of mitochondrial division resulting in an elongated mitochondrial network might thus represent a potential therapeutic approach in many malignancies. Accordingly, the quinazolinone derivative known as mitochondrial division inhibitor (mdivi-1) acts as a Drp1 inhibitor that blocks mitochondrial fission [35]. Virtual screening of a chemical library identified an ellipticine compound, named Drpitor1, as a putative Drp1 inhibitor, which was chemically modified to remove the methoxymethyl group and reduce its hydrolytic lability, obtaining the compound Drpitor1a. Both Drpitor1 and Drpitor1a inhibited the GTPase activity of Drp1 without affecting the GTPase of dynamin 1, and had a greater potency than mdivi-1; both compounds also reduced proliferation and induced apoptosis in cancer cells in vitro as well as in vivo in a xenograft model of lung cancer [36].

In prostate cancer cells, MFF interacts with the voltage-dependent anion channel-1 (VDAC1) at the OMM, and blocking such interaction results in a collapse of mitochondrial functions with increased MOMP, loss of inner membrane potential, Ca^2+^ unbalance, bioenergetics defects, and activation of cell death pathways [37]. The MFF–VDAC1 complex acts therefore as a novel regulator of mitochondrial cell death and appears a promising actionable therapeutic target in cancer, as demonstrated by the findings that an MFF peptidomimetic was well-tolerated and elicited anti-cancer activity in several preclinical models, including patient-derived xenografts, primary breast and lung adenocarcinoma organoids, and glioblastoma neurospheres [38].

The metastatic capacity of breast cancer cells could also be dampened by silencing Drp1, or by Mfn1 overexpression, promoting mitochondrial elongation [21]. Furthermore, proteins associated with mitochondrial fusion, such as Mfn1/Mfn2 and Opa1, have been recently reported as essential players for tumor angiogenesis [39] as they increase the oxygen consumption and cellular ATP production in liver cancer cells [40]. The knock-down of Opa1 in ovarian tumor cells promoted disarrangement of cristae and fragmentation of the mitochondrial network, which was associated with the dissipation of the mitochondrial transmembrane potential, cytochrome *c* release, and apoptosis [41]. 

The population of stem cells in cancer (CSCs) generally show a significant increase in mitochondrial mass, hyperfused mitochondria, and higher mRNA expression of genes involved in mitochondrial biogenesis. Interestingly, the protein Opa1 was found highly expressed in pancreatic ductal adenocarcinoma (PDAC)-derived SCs and positively modulated their propagation in vitro [42]. A signature of Opa1 upregulation has been reported in triple-negative breast cancer (TNBC), correlating with worse prognosis. In TNBC cells, Opa1 inhibition antagonized proliferation, migration, and invasion in vitro and in vivo. Mechanistically, while Opa1 silencing did not affect mitochondrial respiration, it upregulated the tumor suppressors of the miR-148/152 family [43]. Increased mitochondrial biogenesis and Opa1-mediated alterations in cristae morphology were associated with chemoresistance [44,45,46]. Moreover, the formation of a large fused mitochondrial network in response to chemotherapeutic drugs, with upregulation of Mfn1 and Mfn2 genes, has been described in paclitaxel-resistant lung adenocarcinomas and other chemoresistant settings [47]. Noteworthy, enhanced mitochondrial fission in PDAC could be reversed by overexpression of Mfn2 or by its pharmacologic reactivation with leflunomide, an oral FDA-approved anti-arthritis drug which could be likely repurposed as chemotherapeutic agent in this cancer [48].

## 4. Non-Coding RNA-Regulation of Mitochondrial Dynamics

Mitochondria are key cellular energy source, and changes in mitochondrial dynamics alter stability and integrity of these organelles, ultimately affecting the fate of cancer cells. A growing body of literature suggests that the fission/fusion unbalance is tumor type-dependent and reflects the growth factor stimuli, genetic makeup, tumor microenvironment, and chemotherapy responsiveness of tumor cells [49,50]. Additionally, it is clearly emerging that mitochondrial dynamics can be finely tuned by non-coding RNAs (ncRNAs) [51]. 

### 4.1. Non-Coding RNA Classification and Function

In humans, ncRNAs represent 70% of the genome and have several regulatory and structural functions. They can be roughly divided into two groups based on their length: long ncRNAs (>200 nt), that include linear and circular RNAs, and small ncRNAs (<200 nt), including, among others, microRNAs (miRNAs), short interfering RNAs (siRNAs), and piwi-interacting RNAs (piRNAs) [51,52].

MicroRNAs (miRNAs) are a class of 22-nucleotide-long ncRNAs which carry out their canonical function in the cytoplasm by binding the 3′-UTR of a hundred target mRNAs, resulting in the inhibition or degradation of their translation. They are considered master regulators of physiological processes and play a pivotal role in cancer progression, acting as oncogenes or tumor suppressors [53,54].

Several nuclear-encoded miRNAs have been found to impact mitochondria and indirectly affect their biology, for instance through the targeting of the mRNAs encoding proteins of the OXPHOS system [52] or mitochondrial transporters and carrier proteins [55]; moreover, miRNAs are connected to amino acids and nucleotide metabolism in mitochondria, or involved in mitochondria-mediated apoptosis, autophagy, and mitophagy [56].

Based on their localization and origin, mitochondrial ncRNAs can be distinguished into two groups: (1) nuclear-encoded RNAs, involved in anterograde communication as a nuclear contribution to the mitochondrial transcriptome; (2) mitochondria-localized ncRNAs (encoded in the nucleus or mitochondria), involved in retrograde signals and exported to the cytoplasm where they act. Mitochondria-localized ncRNAs, named mitomiRs, are a class of miRNAs with mitochondrial localization which regulate gene expression and function. Most mitomiRs originate from the nuclear genome and are imported into the mitochondria, but there is also evidence of mtDNA-encoded miRNAs [57,58,59]. 

miRNAs have been also proven to act on mitochondria by impairing the process of ROS removal, thus suppressing tumorigenesis of various cancers, as well as by increasing apoptosis by targeting the mitochondrial antioxidant machinery and accumulating oxidative stress; some mitomiRs trigger apoptosis by inducing mitochondrial depolarization and mitochondrial fragmentation.

LncRNAs represent the largest portion of nuclear non-coding transcriptome in humans, and are involved in several functions, including transcriptional regulation, organization of nuclear domains, protein scaffolding, and miRNAs sponging. LncRNA alteration has been associated with different disease states [60]. In tumor cells, several lncRNAs, including both mitochondria- and nuclei-encoded molecular species, were found to directly or indirectly modulate mitochondria reprogramming. To increase biosynthetic and reduce bioenergetic pathways, cancer cells must compensate the lower ATP production afforded by glycolysis respect to OXPHOS, thus reprogramming energy metabolism [61,62]. Nuclear-encoded lncRNAs are transported into the mitochondria to monitor mitochondria status, morphology, and function. These lncRNAs can affect mitochondria metabolism and apoptosis by interacting with proteins implicated in mitochondrial metabolism and translation of mitochondrial-encoded peptides [63]. Nuclear-encoded lncRNAs have been also implicated in the interaction with mitochondria metabolism-related proteins, impacting on mitochondria function and tumor angiogenesis. MALAT1 is the most characterized nuclear-localized lncRNA found overexpressed in different malignancies [64], acting as a critical epigenetic player in the regulation of mitochondrial metabolism of hepatocarcinoma cells, and whose knock-down induces multiple abnormalities in mitochondria, including altered structure, low oxidative phosphorylation (OXPHOS), decreased ATP production, reduced mitophagy, decreased mtDNA copy number, and activation of mitochondrial apoptosis [63]. Mitochondria are also capable of transcribing their own lncRNAs from the mtDNA.

**Figure 2:**
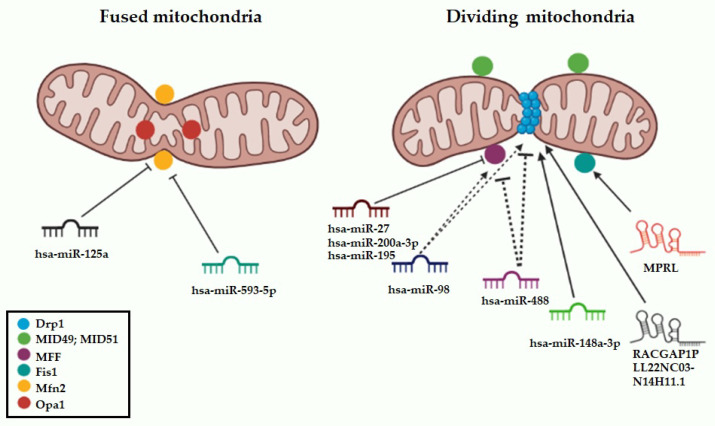
Graphical overview of miRNAs and long non-coding RNAs regulating mitochondrial dynamics targets. Blunted arrow indicates negative regulation through 3′ UTR targeting, regular arrows indicate positive regulation of the mitochondrial target; dashed lines indicate non-canonical targeting. The picture was created using BioRender software.

Many authors have demonstrated growing interest in the comprehension of the molecular mechanisms by which ncRNAs affect mitochondrial dynamics and contribute, directly or indirectly, to specific cancer hallmarks. Such ncRNAs will be discussed in the following sections, as well as graphically depicted in Figure 2 and summarized in Table 1.

### 4.2. miRNAs Targeting Mitochondrial Dynamics Effectors

#### 4.2.1. *hsa-miR-125a*

Dysregulation of miRNAs’ fragile genomic regions has been frequently associated with human cancer onset and progression. miR-125a is located at the 19q13 chromosome, which is frequently deleted in several human cancers, and may function as a tumor suppressor during carcinogenesis [79]. This miRNA has been shown to inhibit proliferation of gastric cancer, lung cancer, hepatocellular carcinoma, as well as ovarian cancer [79,80,81,82]. 

Recent studies highlighted the tumor-suppressive role of miR-125a also in pancreatic cancer, where this miRNA acts by affecting mitochondrial dynamics and modulating cellular apoptosis, energy metabolism, and migration. By comparing miR-125a mimic and inhibitor activity in pancreatic cancer cells, the authors showed that miR-125a acts as pro-apoptotic factor and mitochondrial fission activator; accordingly, the inhibition of miR-125a led to an increase in mitochondria number with a longer length compared to control. On the contrary, miR-125a mimic markedly enhanced fragmented mitochondria, and this effect was abolished by the co-treatment with mdivi-1, a mitochondrial fission inhibitor. Ectopic miR-125a-dependent mitochondrial fragmentation impaired mitochondrial ATP production by reducing ETC activity, resulting in the reduction in cancer cell survival and migration via F-actin depolymerization into G-actin. Mitochondrial homeostasis is largely dependent on Mfn2 function, which preserves fusion/fission balance and controls Drp1 activity. The authors demonstrated an inverse correlation between Mfn2 and miR-125a levels in pancreatic cancer cells; moreover, Mfn2 was validated as a miR-125a direct target, and as a consequence, miR-125a replacement triggered excessive mitochondrial fission via its downregulation, leading to activation of mitochondria-associated apoptosis, with MOMP dissipation and subsequent cytochrome *c* leakage. Importantly, the miR-125a/Mfn2 axis was found to be regulated by hypoxia-inducible factor 1 (HIF1); accordingly, hypoxic conditions further repressed miR-125a levels, resulting in the reduction in mitochondrial fission and increasing tumor progression [65].

#### 4.2.2. *hsa-miR-148a-3p*

miR-148a-3p belongs to the miR-148/152 family and has been established as a tumor-suppressor miRNA in several cancer types [83,84,85], including gastric cancer [86]. The reconstitution of miR-148a-3p, by using miRNA mimic, sensitized gastric cancer cells to cisplatin cytotoxicity by triggering mitochondria-mediated apoptosis and reducing colony formation. These effects were related to the acquisition of features typical of mitochondrial fission and dysfunction, as the decrease in mitochondrial membrane potential and triggering of ROS. Moreover, miR-148a-3p reconstitution dramatically decreased, whereas miR-148a-3p repression promoted, LC3-II protein levels, promoting cisplatin-induced apoptosis through the inhibition of autophagosome formation. Supporting the potential link between miR-148a-3p and mitochondrial dysfunction, the authors showed that miR-148a-3p downregulation in cisplatin-resistant gastric cancer cells was accompanied by upregulation of AKAP1, a direct miR-148a-3p downstream target, which antagonized cisplatin-induced mitochondrial fission, promoting PKA-dependent Drp1 phosphorylation at S637. In this scenario, AKAP1 worked as a molecular scaffold on the OMM, protecting Drp1 from cisplatin-induced activation, without affecting Drp1 expression. Moreover, low miR-148a-3p levels were found to correlate with upregulation of the member of the Ras oncogene family RAB12, another miR-148a-3p target, known as an autophagy inducer. Finally, through decreasing AKAP1 and RAB12 expression levels, lentiviral-mediated miR-148a-3p overexpression sensitized gastric cancer xenografts to cisplatin treatment in vivo [66].

#### 4.2.3. *hsa-miR-98*

miR-98 belongs to the let-7 family of miRNAs and is associated with various types of human cancers, in which its biological role seems controversial. It has been found overexpressed in head and neck squamous carcinoma and breast cancers [87,88], while downregulated in other types of tumors [89]. The role of miR-98 is also critical for the tumorigenesis of bladder cancer; specifically, miR-98 was upregulated in bladder cancer tissues compared to normal matched samples, without any clear correlation with tumor grade or invasion. In this context, miR-98 acts as oncogenic miRNA, promoting cancer cell proliferation, inhibiting apoptotic pathways, and inducing multidrug resistance via mitochondrial dynamics modulation. The authors explored the biological function of miR-98 by using both miR-98 gain and loss-of-function, observing that ectopic expression of miR-98 accelerated cell growth and colony formation ability and reduced cisplatin sensitivity. On the other hand, the transfection of an antagomiR-98 led to a reduction in cancer cell viability, increasing apoptosis, and overcoming cisplatin resistance [67]. Different studies underscore a potential relationship between mitochondrial function and chemotherapy resistance [90]. Further investigation unraveled the role of miR-98-related cisplatin resistance and mitochondrial dynamics, demonstrating that ectopic miR-98 was able to promote mitochondrial fragmentation in cancer cells, with the upregulation of active Drp1 and its receptor Fis1, and increase in mitochondrial membrane potential, resulting in chemotherapy resistance. Conversely, targeting of miR-98 by a miR-98 inhibitor promoted mitochondrial fusion, sensitizing cancer cells to cisplatin-induced apoptosis, with downregulation of Drp1 phosphorylation at S616. Interestingly, miR-98 inhibitor promoted the overexpression of the tumor suppressor LASS2, a miR-98 direct target, which reduced mitochondrial membrane potential and increased mitochondrial fusion via Drp1 inhibition [67].

#### 4.2.4. *hsa-miR-29c-3p*

As remarked above, cancer cells regulate mitochondrial dynamics to meet their metabolic requirements for the development and progression of cancer. An important role as mitochondrial fission regulator is played by MTFR1, a mitochondrial protein found overexpressed in lung cancer cell lines and tissues, and positively correlated with adverse clinicopathological features as well as poor overall survival of cancer patients. Upregulation of MTFR1 by lentiviral-mediated overexpression affected the mitochondrial dynamics balance in lung cancer cells, increasing mitochondrial fission and leading to stimulation of proliferation, invasion, and migration of cancer cells both in vitro and in vivo in a xenograft model. High MTFR1 levels inhibited apoptosis, as confirmed by a reduction in PARP and BAX proteins. Interestingly, overexpression of MTFR1 promoted aerobic glycolysis by increasing glucose consumption and lactate production, exerting its oncogenic function by targeting the AMPK/mTOR signaling pathway in lung cancer cells. Based on bioinformatic analysis, the authors predicted miR-29c-3p as upstream putative regulator of MTFR1, subsequently validated as direct target by reporter assays [68]. miR-29c-3p belongs to the miR-29 family [91] and is widely expressed in both healthy and tumor tissues. It may be involved in cancer progression, metastasis, and drug resistance in ovarian cancer as well as hepatocellular carcinoma [92,93]. 

Replacement of miR-29c-3p suppressed the progression of different cancer types, such as cervical, ovarian, and esophageal cancers [94,95]. miR-29c-3p expression was found downregulated in lung cancer cells and inversely correlated to MTFR1 levels. miR-29c-3p replacement through miRNA mimics led to downregulation of MTFR1 both at mRNA and protein levels, resulting in a partial rescue of the phenotypic changes induced by MTFR1 on cell proliferation, migration, and invasion. Accordingly, elevated miR-29c-3p levels accounted for the inhibition of MTFR1-dependent glycolytic capacity, and overall reduced the expression of proteins associated with cell proliferation and EMT [68].

#### 4.2.5. *hsa-miR-593-5p*

miR-593-5p, an intronic miRNA within the SND1 host gene, has been found downregulated in different types of human malignant tumors, including gastric, esophageal, tongue, lung, and breast cancers, where its overexpression inhibits tumor proliferation and metastasis [96,97]. It has been reported that the activation of mitochondrial fission is induced by cisplatin treatment, in tongue squamous cell carcinoma (TSCC), as a precursor event of apoptotic cell death. Upon cisplatin stimulation, Fan et al. evidenced MFF upregulation at protein level, whereas no significant change at mRNA level was detectable, suggesting that MFF is potentially regulated at post-transcriptional level by miRNAs. Indeed, bioinformatics analysis highlighted binding sites for miR-593-5p within the 3′ UTR of MFF, which were validated by reporter assays. Interestingly, cisplatin exposure induced downregulation of miR-593-5p and MFF upregulation, resulting in the activation of mitochondrial fragmentation and apoptotic cell death. By contrast, the enforced expression of miR-593-5p by miRNA mimic attenuated MFF protein levels, even after cisplatin exposure, thus triggering drug resistance through inhibition of mitochondrial fission.

To explore the mechanisms underlying cisplatin-dependent deregulation of miR-593-5p, the authors investigated the miR-593-5p transcriptional regulation, analyzing a 5-kb upstream region of miR-593-5p, and identifying putative binding sites for BRCA1, suggesting the involvement of this tumor suppressor in the positive regulation of miR-593-5p. Accordingly, BRCA1 was downregulated by cisplatin, and its silencing or enforced expression led to miR-593-5p down- or upregulation, respectively. In addition, exogenous BRCA1 attenuated cisplatin-induced downregulation of miR-593-5p, with an inhibitory effect on mitochondrial fission and apoptosis induction in tumor cells. A key role of the BRCA1/miR-593-5p/MFF axis, with functional implications in mitochondrial fission and cisplatin sensitivity, was also established in vivo in different TCSS-xenografted murine models, where the knockdown of miR-593-5p decreased the negative effect of BRCA1 on mitochondrial apoptosis and fission, whereas the overexpression of this miRNA led to an increase in tumor growth, even in the presence of cisplatin. Clinically, the levels of components of BRCA1/miR-593-5p/MFF axis significantly correlated with cisplatin sensitivity and the overall survival of TSCC patients [69].

#### 4.2.6. *has-miR-27* and *hsa-miR-200-3p*

miR-200-3p (chromosome 1p36.33) belongs to the miR-200 family, which consists of five members, divided according to their location in the gene cluster, classified into two groups (miR-200a/b/429 and miR-200c/141), which have oncogenic or tumor-suppressive effects. miR-200a-3p is strictly related to multiple types of cancer and regulates their onset and progression, likely by affecting cell proliferation and epithelial-to-mesenchymal transition pathways [98].

miR-27a is transcribed from the miR-27a gene on chromosome 19p13.13; due to its role in promoting cell proliferation, chemotherapy resistance, cell metabolism alteration, tumor immune response, and epithelial-mesenchymal transition [99], it was suggested to be an onco-miRNA in various cancers [100]. 

Both miR-200a-3p and miR-27 were found to target the 3′UTR of MFF, promoting mitochondrial elongation, suggestive of enhanced fusion, leading to increased mitochondrial ATP synthesis and membrane potential [70,71].

Subsequently, the regulatory role of both miRNAs on mitochondrial dynamics was shown to depend on their negative activity on the T-cell-restricted intracellular antigen 1 (TIA-1), known to upregulate MFF in HCC cells by directing promoting its mRNA translation [72]. In silico and wet analyses demonstrated the upregulation of the expression levels of both TIA-1 and MFF in HCC cell lines, as well as in tissues derived from HCC patients, clearly correlating with poor survival rates of patients. Moreover, sera of HCC patients displayed an increase in both TIA-1 and MFF levels, which was also observed in an in vitro model of liver cancer. By comparing HCC biopsies with non-cancerous adjacent tissues, miR-200a-3p and miR-27a/b were found significantly downregulated in cancer specimens, suggesting an inverse correlation between TIA-1/MFF mRNAs and miR-200a-3p/miR-27a, and underscoring their potential as novel biomarkers in HCC [73].

#### 4.2.7. *hsa-miR-488*

miR-488 plays a role in various human diseases, including cancer. It acts as a tumor suppressor promoting apoptosis and inhibiting proliferation, cell cycle progression, colony formation, and cell migration in different types of cancers, including non-small cell lung cancer (NSCLC), prostate, gastric, and ovarian cancers [101,102,103]. Notably, enforced expression of miR-488, by using a synthetic miRNA mimic, reduced ovarian cancer cell proliferation and colony formation units, while increasing apoptosis. These effects were more evident when ovarian cancer cells were co-treated with miR-488 mimic and cisplatin combination. Since cisplatin has been reported to induce mitochondrial dysfunction, cytochrome *c* release, and the production of mitochondrial superoxide species in drug-sensitive ovarian cancer cells, the authors hypothesized that unbalanced mitochondrial dynamics might be implicated in chemotherapy resistance. Accordingly, a mitochondrial morphological examination revealed the presence of elongated mitochondria in ovarian cancer cell lines treated with miR-488 mimic, with a decreased mitochondrial membrane potential and a downregulation of the main mitochondrial fission regulators, Drp1 and Fis1. Further analysis validated the direct targeting—and the inverse correlation with miR-488 levels—of Sine oculis homeobox homolog 1 (Six1), an oncoprotein likely responsible of drug resistance and mitochondrial regulation, that acted through positive regulation of Drp1 phosphorylation and mitochondrial fission. miR-488 replacement induced a downregulation of Six1 and consequently affected Drp1 signaling to regulate mitochondrial function and chemoresistance [74].

#### 4.2.8. *hsa-miR-195*

Low miR-195 expression was observed in different tumors and found correlated with advanced clinical stage, metastasis, and chemoresistance [104,105]. Additionally, miR-195 was found differentially expressed in normal and breast cancer tissues [106], and its downregulation in tumor cells ascribed to CpG promoter hypermethylation [107]. miR-195 is one of the members of the miR-15/107 family and its overexpression induced pro-apoptotic, non-invasive, and anti-metastatic effects in breast cancer. Purohit et al. demonstrated miR-195 involvement in mitochondrial inner membrane depolarization and alteration of mitochondria metabolic function. Specifically, the authors observed miR-195 acting as a modulator of mitochondrial dynamics; accordingly, the miR-195 replacement induced features of mitochondrial fission via direct targeting of MFN2 3′UTR, as well as increasing Drp1 levels, thereby affecting mitochondrial morphology and activity in breast cancer cells. Moreover, in vitro miR-195-induced tumor-suppressive effects were accompanied by mitochondrial membrane potential depolarization and reduced active mitochondrial mass, without affecting total mass or inducing mitophagy as repair mechanism. Moreover, miR-195 overexpression affected mitochondrial respiration by decreasing oxygen consumption rate and ATP production, and increasing oxidative stress, while maintaining mitochondrial superoxide concentration at non-damaging levels through upregulation of the main scavenger protein MnSOD2 [55].

### 4.3. lncRNAs Targeting Mitochondrial Dynamics Effectors

#### 4.3.1. *RACGAP1P*

Pseudogenes are nucleotide sequences derived from ancestral genes lacking the ability to be expressed. RACGAP1P is the pseudogene of RACGAP1, acting as a lncRNA with oncogenic functions in different cancer types. This lncRNA is highly expressed in HCC and breast cancer, promotes cell proliferation and migration in vitro and in vivo, and its elevated expression predicts poor prognosis of patients with HCC [108]. Mechanistically, RACGAP1P has competing endogenous RNAs (ceRNA) activity, competing with miR-15-5p by inhibiting the interactions with its mRNA targets, activating RhoA/ERK signaling, and contributing to the relapse of HCC. The close correlation between lncRACGAP1P and tumor metastases at different sites and lymph node involvement, along with the emerging role of mitochondrial fragmentation in the migration process, led the authors to investigate the link between RACGAP1P and mitochondrial fission in the promotion of breast cancer metastasis. They found that lncRACGAP1P induced mitochondrial fission, through the increase in phosphorylation of Drp1 on S616. Moreover, by evaluating both the migratory activity and mitochondrial morphology in different breast cancer cell models, the authors demonstrated that mitochondrial fission is elevated in those cell lines with high invasive capacity compared to the less invasive ones. In addition, Drp1 pharmacologic inhibition by mdivi-1 led to a shift from mitochondrial fission towards fusion, followed by a reduction in invasion of RACGAP1-overexpressing breast cancer cells compared to the control; notably, treatment with M1, a promoter of mitochondrial fusion, led to the same phenotypic effects, suggesting that overexpression of RACGAP1P promoted mitochondrial fission, which is necessary for the invasion of breast cancer cells [75].

#### 4.3.2. *MPRL*

miRNA processing-related lncRNA NR_034085 (MPRL) is a lncRNA, located on the human chromosome 5 and consisting of an exon with a total length of 2869 nt, involved in miRNA processing because of its DICER-inhibiting activity; it is transactivated by E2F1 transcription factor and has a putative tumor-suppressor activity. Recently, its role in promoting apoptosis, mitochondrial fission, and sensitivity to cisplatin in tongue squamous cell carcinoma (TSCC) has emerged [76]. Cisplatin is a well-known chemotherapeutic agent that induces apoptosis via the release of cytochrome *c* and activation of the caspase cascade [109]. The lncRNA MPRL interacts in the cytoplasm with pre-miR-483 with a complementarity of 19 nt at the DICER binding and clipping sites, blocking the formation of mature miR-483-5p. MPRL participates in the regulation of mitochondrial dynamics, particularly in cisplatin-treated TSCC cells. Indeed, miR-483-5p antagonized cisplatin sensitivity and inhibited mitochondrial fission of TSCC cells by binding to its main target Fis1; conversely, the over-expression of MPRL led to an increase in mitochondrial fission by blocking the formation of miR-483-5p. Importantly, silencing of MPRL dampened mitochondrial fission, decreasing apoptosis, and increasing resistance to chemotherapy. Additionally, increased expression of miR-483-5p was associated with poor prognosis in different cancer types. Thus, there is a negative correlation between the two ncRNAs: forced MPRL expression reduces pre-miR-483 levels, as it masks the recognition of pre-miR-483 by TRBP-DICER, leading to inhibition of tumor growth, increased cisplatin sensitivity, and mitochondrial fragmentation [76].

#### 4.3.3. *LL22NC03-N14H11.1*

By interrogating the GEPIA database, the long non-coding LL22NC03-N14H11.1, located on chr22:16,154,073-16,154,766, was found upregulated in HCC and associated with poor prognosis of HCC patients, suggesting a potential oncogenic role. This lncRNA showed higher levels in metastatic than non-metastatic HCC patients; LL22NC03-N14H11.1 was also found upregulated in HCC cell lines respect to normal control cells, with a preferential localization in the nucleus than in the cytoplasm. LL22NC03-N14H11.1-based loss-of-function strategies led to a decrease in cell viability and colony formation ability of HCC cell lines, antagonizing EMT and increasing apoptosis. Conversely, gain-of-function of LL22NC03-N14H11.1 strengthened the viability, migration, and invasion of HCC cells. Of note, LL22NC03-N14H11.1 silencing produced elongated and hyperfused mitochondria, whereas the opposite phenomenon was observed after lncRNA overexpression, underscoring an effect on mitochondrial dynamics. Consistently, LL22NC03-N14H11.1 overexpression boosted, whereas its downregulation reduced, Drp1 phosphorylation on S616, thus confirming the involvement of LL22NC03-N14H11.1 in the positive regulation of mitochondrial fragmentation; in line with this effect, Drp1 overexpression abrogated the negative effects on cell proliferation triggered by LL22NC03-N14H11.1 antagonism, reducing the activation of apoptotic pathways, while restoring invasion and migration of HCC cell lines. Mechanistically, it was demonstrated that LL22NC03-N14H11.1 increased phosphorylated ERK1/2, a key positive regulator of Drp1 activation via phosphorylation at S616, and also inhibited the ubiquitination of H-RAS (G12V) mediated by LZTR1, a tumor-suppressor known to repress the MAPK pathway.

At a molecular level, LL22NC03-N14H11.1 repressed in the nucleus the transcription of LZTR1 by recruiting c-Myb transcription factor to its promoter, leading to the activation of the H-RAS/MAPK pathway. Accordingly, the knockdown of LZTR1, or overexpression of H-RAS, restored the proliferation of LL22NC03-N14H11.1-silenced HCC cells as well as the attenuated mitochondrial fission, suggesting that LL22NC03-N14H11.1 exacerbates the malignant phenotype of HCC cells through the targeting of the LZTR1/H-RAS/MAPK pathway [77].

#### 4.3.4. *CDK6-AS1*

CDK6-AS1 is a lncRNA closely associated with tumor development and progression in acute lymphoblastic leukemia as well as in human gastric epithelial tumors [110,111]. By retrospectively analyzing CDK6-AS1 expression in a cohort of 132 pediatric patients harboring several genetic abnormalities, elevated expression of CDK6-AS1 was detected in AML samples as compared to healthy cord-blood derived mononuclear cells. To address the biological role of this lncRNA, the authors first subdivided patients into clinically relevant groups according to either the expression levels of CDK6-AS1 or the genetic abnormalities identified in AML at diagnosis. Notably, AML patients with the highest CDK6-AS1 levels were at high risk of not achieving complete remission, and also had elevated expression of mitochondrial translation-related genes, while being negatively enriched in genes regulated by RUNX1, an essential factor for the early hematopoietic differentiation process. Functionally, it was proven that CDK6-AS1 depletion promoted the differentiation of CD34^+^ cells, demonstrating no effect on cell viability, but inducing a reduction in the colony-forming capacity of AML cells [78].

It is well known that cancer cells modulate their metabolic program toward cellular energy production, inducing drug resistance which strengthens tumor aggressiveness [26]. In this context, high CDK6-AS1 levels activated a transcriptional program to increase mitochondrial mass and membrane potential in leukemia cells, reducing drug sensitivity. Interestingly, AML CD34^+^ with low mitochondrial membrane potential were more quiescent, and mainly CD38^−^ with respect to the CD34^+^ with high mitochondrial membrane potential, which appeared more proliferating and mostly CD38^+^ cells. AML-CD34^+^ showed higher CDK6-AS1 levels and mitochondria content than the more differentiated CD34^−^ AML blasts, confirming that CDK6-AS1 overexpression positively correlated with mitochondrial biogenesis and mass in leukemia cells, especially in the CD34^+^ subpopulation. Overall, these findings suggest that aberrant expression of CDK6-AS1 maintains more immature leukemic stem cell subsets with elevated mitochondrial activities to address high energy demand, inducing drug resistance and apoptosis escape. CDK6-AS1 expression, correlating with mitochondrial status, could thus allow to discriminate AML patients benefitting from the combination of chemotherapy with mitochondria-targeting agents [78].

## 5. Conclusions

Recent advances have demonstrated the link between mitochondrial dynamics and several cancer hallmarks, including proliferation, cell death, and metabolic reprogramming; increasing evidence also underscores the pivotal role of mitochondrial fission in chemoresistance. As discussed in this review, many studies have been carried out to unveil the physiological activity of the mitochondrial fusion/fission machinery, and its alterations in the context of cancer pathophysiology. The vast majority of these studies converge in a pro-oncogenic role of mitochondrial fission, as demonstrated by the therapeutic activity of Drp1 inhibitors or Mfn2 activators in preclinical models, although it also emerges that several cancer-related factors (genetic, metabolic, environmental) can affect the mitochondrial dynamics rheostat and its tumor modulating properties in a tissue-dependent manner [12,14].

Since mitochondrial fusion and fission represent two opposing systems, their balance and role in cell fate are carefully regulated by specific cellular metabolic needs. Therefore, more insights into the regulatory patterns orchestrating mitochondrial dynamics are mandatory for a full elucidation of its role and eventually to develop targeted therapies [9]. In this context, increasing attention has been given to ncRNAs, which are closely linked to mitochondrial functions by regulating the expression of several mitochondrial dynamics effectors.

Non-coding RNAs (ncRNAs) are endogenous RNAs accounting for the majority of the transcribed genome. Initially designated “dark matter” because of their lack of ability to encode proteins, they are currently considered important regulatory molecules in the fine-tuning of key cellular pathways. With the unraveling of the mechanisms by which ncRNAs affect diseases and the development of systems to modulate ncRNA levels both in vitro and in vivo, the feasibility of RNA therapeutics has rapidly expanded [54,112,113,114,115]. As summarized in Table 1, various classes of ncRNAs have been found to regulate mitochondrial dynamics at different levels, impacting on cell metabolism, energy production, and mitochondrial homeostasis, and ultimately affecting survival and chemoresistance of tumor cells.

The limited armamentarium of compounds to selectively inhibit the GTPases effectors of the mitochondrial dynamics machinery indeed makes strategies targeting upstream ncRNA regulators as an alternative and feasible approach to treat tumors with aberrant mitochondrial dynamics. In this regard, the novel emerging strategies of miRNA/lncRNA inhibition, as the new generation antisense oligonucleotides (ASOs), and the possibility to enclose them within functionalized delivery systems to selectively reach the tumor sites [54,112], provide new avenues for targeting undruggable proteins, including those devoid of accessible tasks, or containing widely conserved domains as the mitochondrial dynamics GTPases. It is tempting to speculate that the current advancements in ncRNA knowledge, in terms of both expression profiling and delivery approaches, will disclose important clues on the biological role and therapeutic potential of novel and poorly investigated ncRNA-regulated pathways underlying cancer pathophysiology, including mitochondrial dynamics.

## Figures and Tables

**Table 1:** Cancer-associated ncRNAs targeting mitochondrial dynamics effectors.

Non-Coding RNA	Cancer Type/ Expression	Mitochondrial Dynamics Target	Phenotypic Effects	References
*hsa-miR-125a*	Downregulated in pancreatic cancer	MFN2	miR-125a decreases cancer cell survival and migration	[65]
*hsa-miR-148a-3p*	Downregulated in gastric cancer	AKAP1	miR-148a-3p inhibits gastric cancer cell growth, induces mitochondrial apoptosis, and increases cisplatin sensitivity	[66]
*hsa-miR-98*	Upregulated in bladder cancer cells	LASS2	miR-98 promotes cancer cell proliferation, inhibits apoptosis, and induces multidrug resistance	[67]
*hsa-miR-29c-3p*	Downregulated in lung adenocarcinoma	MTFR1	miR-29c-3p suppresses proliferation, migration, and invasion of lung cancer cells	[68]
*hsa-miR-593-5p*	Downregulated in tongue squamous cell carcinoma after cisplatin	MFF	miR-593-5p inhibits apoptotic cell death and decreases cisplatin sensitivity	[69]
*hsa-miR-27 and hsa-miR-200a-3p*	Downregulated in several tumor types	TIA-1, MFF	miR-27 and miR-200a-3p inhibit migration and invasion of cancer cells	[70,71,72,73]
*hsa-miR-488*	Downregulated in ovarian cancer	Six-1	miR-488 inhibits ovarian cancer cell proliferation, induces apoptosis, and increases cisplatin sensitivity	[74]
*hsa-miR-195*	Downregulated in breast cancer	MFN2	miR-195 exerts pro-apoptotic effects in breast cancer cells	[55]
*RACGAP1P*	Upregulated in breast cancer	Drp1	RACGAP1P promotes breast cancer cell proliferation and metastatic behaviour	[75]
*MPRL*	Downregulated in tongue squamous cell carcinoma	Fis1	MPRL inhibits tumor growth in TSCC cells, activates apoptosis, and induces cisplatin sensitivity	[76]
*LL22NC03-N14H11.1*	Upregulated in hepatocarcinoma	Drp1	LL22NC03-N14H11.1 promotes cell survival, migration, and invasion of HCC cells	[77]
*CDK6-AS1*	Upregulated in acute myeloid leukemia	−	CDK6-AS1 inhibits myeloid differentiation, and increases stemness, drug resistance, and tumor aggressiveness	[78]

## Data Availability

The data presented in this study are available on reasonable request from the corresponding author.
